# An endogenous cholinergic system controls electrical conduction in the heart

**DOI:** 10.1093/eurheartj/ehae699

**Published:** 2024-10-22

**Authors:** Duanyang Xie, Ke Xiong, Nianguo Dong, Guanghua Wang, Qicheng Zou, Beihua Shao, Zhiwen Chen, Luxin Wang, Yu Kong, Xu Wang, Xuling Su, Wenli Bai, Jian Yang, Yi Liu, Bin Zhou, Yi-Han Chen

**Affiliations:** State Key Laboratory of Cardiovascular Diseases, Shanghai East Hospital, School of Medicine, Tongji University, Shanghai 200120, China; Shanghai Arrhythmia Research Center, Shanghai East Hospital, School of Medicine, Tongji University, Shanghai 200120, China; Department of Cardiology, Shanghai East Hospital, School of Medicine, Tongji University, Shanghai 200120, China; Department of Pathology and Pathophysiology, School of Medicine, Tongji University, Shanghai 200092, China; State Key Laboratory of Cardiovascular Diseases, Shanghai East Hospital, School of Medicine, Tongji University, Shanghai 200120, China; Department of Cardiology, Shanghai East Hospital, School of Medicine, Tongji University, Shanghai 200120, China; Department of Cardiovascular Surgery, Union Hospital, Tongji Medical College, Huazhong University of Science and Technology, Wuhan 430022, China; State Key Laboratory of Cardiovascular Diseases, Shanghai East Hospital, School of Medicine, Tongji University, Shanghai 200120, China; Department of Cardiology, Shanghai East Hospital, School of Medicine, Tongji University, Shanghai 200120, China; Department of Pathology and Pathophysiology, School of Medicine, Tongji University, Shanghai 200092, China; State Key Laboratory of Cardiovascular Diseases, Shanghai East Hospital, School of Medicine, Tongji University, Shanghai 200120, China; Department of Cardiology, Shanghai East Hospital, School of Medicine, Tongji University, Shanghai 200120, China; State Key Laboratory of Cardiovascular Diseases, Shanghai East Hospital, School of Medicine, Tongji University, Shanghai 200120, China; Department of Cardiology, Shanghai East Hospital, School of Medicine, Tongji University, Shanghai 200120, China; State Key Laboratory of Cardiovascular Diseases, Shanghai East Hospital, School of Medicine, Tongji University, Shanghai 200120, China; Department of Cardiology, Shanghai East Hospital, School of Medicine, Tongji University, Shanghai 200120, China; Department of Cardiology, Shanghai East Hospital, School of Medicine, Tongji University, Shanghai 200120, China; Electron Microscopy Facilities of Center for Excellence in Brain Science and Technology, Chinese Academy of Science, Shanghai 200031, China; Electron Microscopy Facilities of Center for Excellence in Brain Science and Technology, Chinese Academy of Science, Shanghai 200031, China; Department of Cardiology, Shanghai East Hospital, School of Medicine, Tongji University, Shanghai 200120, China; State Key Laboratory of Cardiovascular Diseases, Shanghai East Hospital, School of Medicine, Tongji University, Shanghai 200120, China; Jinzhou Medical University, Liaoning 121000, China; State Key Laboratory of Cardiovascular Diseases, Shanghai East Hospital, School of Medicine, Tongji University, Shanghai 200120, China; Shanghai Arrhythmia Research Center, Shanghai East Hospital, School of Medicine, Tongji University, Shanghai 200120, China; Department of Cardiology, Shanghai East Hospital, School of Medicine, Tongji University, Shanghai 200120, China; State Key Laboratory of Cardiovascular Diseases, Shanghai East Hospital, School of Medicine, Tongji University, Shanghai 200120, China; Shanghai Arrhythmia Research Center, Shanghai East Hospital, School of Medicine, Tongji University, Shanghai 200120, China; Department of Cardiology, Shanghai East Hospital, School of Medicine, Tongji University, Shanghai 200120, China; Center for Excellence in Molecular Cell Science, Chinese Academy of Sciences, Shanghai 200031, China; State Key Laboratory of Cardiovascular Diseases, Shanghai East Hospital, School of Medicine, Tongji University, Shanghai 200120, China; Shanghai Arrhythmia Research Center, Shanghai East Hospital, School of Medicine, Tongji University, Shanghai 200120, China; Department of Cardiology, Shanghai East Hospital, School of Medicine, Tongji University, Shanghai 200120, China; Department of Pathology and Pathophysiology, School of Medicine, Tongji University, Shanghai 200092, China; Research Units of Origin and Regulation of Heart Rhythm, Chinese Academy of Medical Sciences, Shanghai 200092, China

**Keywords:** Heart, Cholinergic system, Electrophysiology, Excitability, Conductivity, Arrhythmias

## Abstract

**Background and Aims:**

The cholinergic system is distributed in the nervous system, mediating electrical conduction through acetylcholine (ACh). This study aims to identify whether the heart possesses an intact endogenous cholinergic system and to explore its electrophysiological functions and relationship with arrhythmias in both humans and animals.

**Methods:**

The components of the heart’s endogenous cholinergic system were identified by a combination of multiple molecular cell biology techniques. The relationship of this system with cardiac electrical conduction and arrhythmias was analysed through electrophysiological techniques.

**Results:**

An intact cholinergic system including ACh, ACh transmitter vesicles, ACh transporters, ACh metabolic enzymes, and ACh receptors was identified in both human and mouse ventricular cardiomyocytes (VCs). The key components of the system significantly regulated the conductivity of electrical excitation among VCs. The influence of this system on electrical excitation conduction was further confirmed both in the mice with α4 or α7 nicotinic ACh receptors (nAChRs) knockouts and in the monolayers of human induced pluripotent stem cell-derived cardiomyocytes. Mechanistically, ACh induced an inward current through nAChRs to reduce the minimum threshold current required to generate an action potential in VCs, thereby enhancing the excitability that acts as a prerequisite for electrical conduction. Importantly, defects in this system were associated with fatal ventricular arrhythmias in both patients and mice.

**Conclusions:**

This study identifies an integrated cholinergic system inherent to the heart, rather than external nerves that can effectively control cardiac electrical conduction. The discovery reveals arrhythmia mechanisms beyond classical theories and opens new directions for arrhythmia research.

## Structured Graphical Abstract

**Structured Graphical Abstract:**
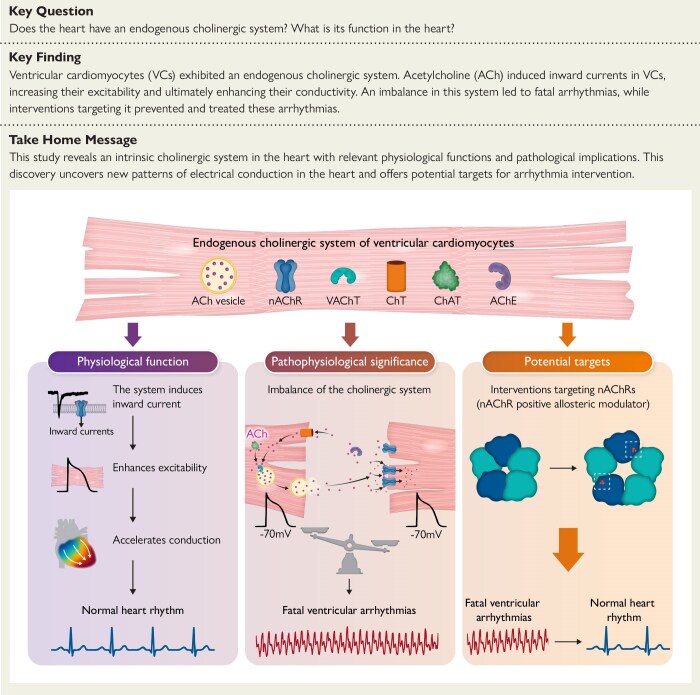
In ventricular cardiomyocytes (VCs), there exists an intrinsic cholinergic system. Within this system, acetylcholine (ACh) acts as a transmitter to induce inward currents through nicotinic ACh receptors (nAChRs), enhancing the excitability of VCs and ultimately accelerating electrical conduction in ventricular myocardium. Defects in this cholinergic system lead to ventricular conduction block and lethal ventricular arrhythmias. Positive allosteric modulators of nAChRs can effectively prevent and terminate lethal ventricular arrhythmias. VAChT, vesicular ACh transporter; ChT, choline transporter; ChAT, choline acetyltransferase; AChE, acetylcholinesterase.

**See the editorial comment for this article ‘Non-neuronal ventricular cardiomyocytelocated nicotinergic acetylcholine receptors cause remodelling and arrhythmias’, by S.F. Noujaim and D. Dobrev, https://doi.org/10.1093/eurheartj/ehae845.**

**Translational perspective**

A well-known issue is that antiarrhythmic drugs face serious challenges: their efficacy is often unsatisfactory, and they can exacerbate existing arrhythmias or induce new ones, including lethal arrhythmias. There is an urgent need for the development of new antiarrhythmic drugs. Currently, the bottleneck in antiarrhythmic drug development is the lack of new effective targets. The study uncovers an intrinsic cholinergic system in the heart. Its imbalance contributes to fatal arrhythmias, and targeted interventions effectively prevent and treat these arrhythmias. These findings provide a novel potential target system containing multiple targets for the development of new antiarrhythmic drugs.

## Introduction

In the nervous system, electrical conduction is mediated by transmitter systems such as the cholinergic system and/or gap junctions.^1–3^ The electrical conduction mediated by the transmitter system is also known as chemical transmission or chemical synaptic transmission.^4^ This type of electrical conduction is the most ubiquitous form of electrical activity in neurons. Importantly, many neurological diseases are related to the abnormalities in the structure and function of the transmitter system, and interventions targeting key elements of these transmitter systems can effectively prevent and treat these diseases.^5,6^ Research has shown that the heart can also partially express components of the transmitter system.^7^ However, whether the transmitter system can act as a whole to regulate the electrical conduction in the heart remains unclear.^8^

Recently, we identified abundant transmitter vesicles under the surface membrane of atrial cardiomyocytes and proved that the atrial cardiomyocytes possess an intact endogenous glutamatergic system that controls the conductivity of the atrial myocardium through a transmitter system-mediated manner. Furthermore, defects in the glutamatergic system are associated with atrial arrhythmias.^9^ Thus, we hypothesized that ventricular electrical conduction may also be controlled by a transmitter system.

In the present study, we reveal that ventricular cardiomyocytes (VCs) possess an intact inherent cholinergic system, with a composition similar to that of the nervous system. This system powerfully controls the conduction of electrical excitation among VCs. Acetylcholine (ACh), through this endogenous cholinergic system, induces an inward current in VCs, reducing the minimum threshold current needed for action potential (AP) formation, thereby increasing the excitability of VCs. The regulation of VC excitability by this system serves as the mechanism for controlling the conduction of electrical excitation. Interestingly, defects in this system are closely associated with life-threatening ventricular arrhythmias both in patients with heart disease and in animal models. Further research has confirmed that interventions targeting key components of this cholinergic system can prevent and terminate life-threatening ventricular arrhythmias. We have discovered a new cardiac bioelectrical control system in the heart, providing a new future for the study of arrhythmia mechanisms and drug development.

## Methods

Methods are described in full detail in the Supplementary material.

## Results

### Functional transmitter vesicles are identified in both human and mouse ventricular cardiomyocytes

**Figure 1:**
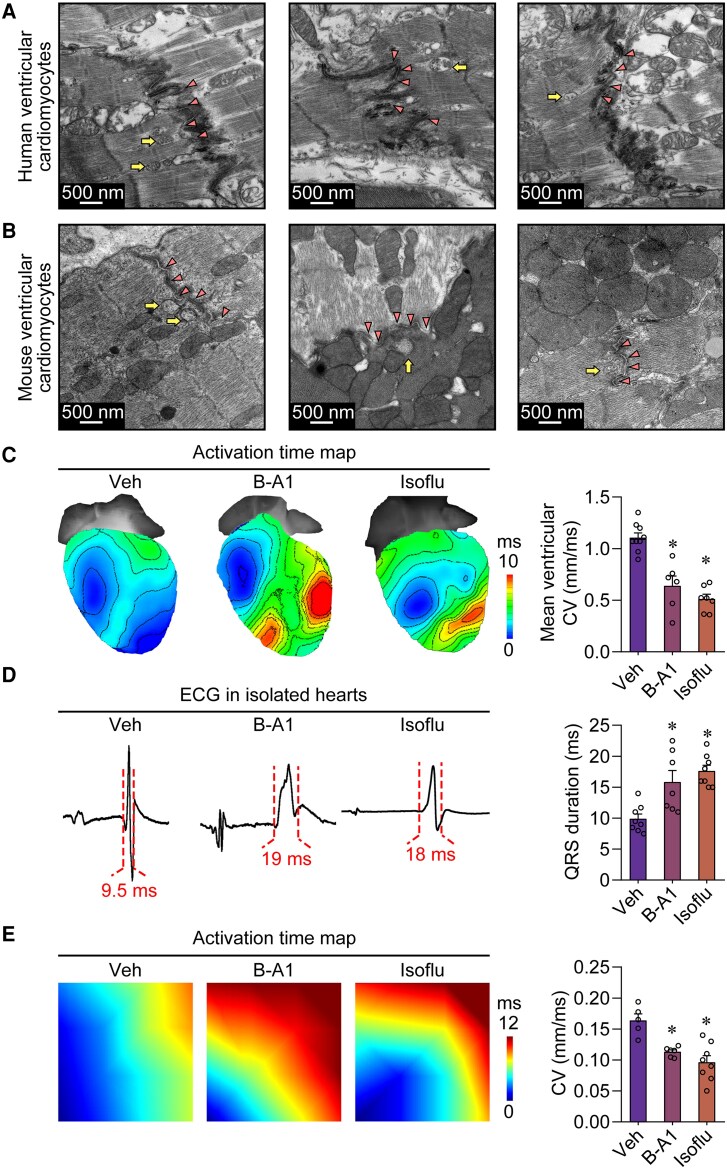
Functional transmitter vesicles in the ventricular cardiomyocytes. (*A*, *B*) Representative transmission electron microscope images showing vesicles (arrows) beneath the plasma membrane of ventricular cardiomyocytes from humans and mice. The triangles indicate the cell–cell junctions, while the angles indicate the vesicles. (*C*) Representative images from optical mapping and the statistical analysis of the conduction velocity in the mouse ventricles in the presence of vehicle, 100 μM bafilomycin A1, or 1 mM isoflurane. *P < .05, calculated by one-way ANOVA; *n* = 6–9 per group. (*D*) Representative electrocardiogram recordings and the statistical analysis of the QRS duration in isolated mouse hearts perfused with vehicle, bafilomycin A1, or isoflurane. *P < .05, calculated by one-way ANOVA; *n* = 7–8 per group. (*E*), Representative images of activation time maps from multi-electrode array recordings and the statistical analysis of the conduction velocity in monolayer of human induced pluripotent stem cell-derived cardiomyocytes treated with vehicle, bafilomycin A1, or isoflurane. *P < .05, calculated by one-way ANOVA; *n* = 5–8 per group. Veh, vehicle; B-A1, bafilomycin A1; Isoflu, isoflurane

Transmitter vesicles are characteristic ultrastructures that mediate chemical transmission-dependent electrical excitation.^1,10,11^ Thus, we first looked for transmitter vesicles in human VCs. Through the transmission electron microscopy, we found transmitter vesicle-like ultrastructures under the surface membrane of human VCs (*Figure 1A*). Detailed analysis revealed that the vesicles appeared as spherical or near-spherical structures and were enclosed by a lipid membrane. We also found similar transmitter vesicles in the mouse VCs, suggesting that the existence of these transmitter vesicles was conserved between human and mouse VCs (*Figure 1B*).

Although we showed that VCs possess transmitter vesicle-like ultrastructures, these structures do not necessarily have physiological functions for the VCs. In the nervous system, transmitter vesicles are involved in the conduction of electrical excitation.^4,10^ Thus, we investigated whether bafilomycin A1 and isoflurane, which can block transmitter release from vesicles in subcellular membranes,^12,13^ could modify the electrical conductivity of Langendorff-perfused mouse hearts, thereby assessing the functionality of these transmitter vesicles. By optical mapping, we found a significant reduction in the ventricular conduction velocity after the use of the two inhibitors, and these results were also confirmed by a significant prolongation of the duration of the Q, R, and S wave (QRS) on electrocardiogram (ECG) (*Figure 1C* and *D*).

In order to rule out the influence of foreign innervation, we seeded the human induced pluripotent stem cell-derived cardiomyocytes (hiPS-CM) onto the probes of multi-electrode array and then observed the effect of the two inhibitors on the electrical conduction of the hiPS-CM monolayer. Consistent with the above results on the isolated perfused heart, the electrical conduction velocity within hiPS-CM monolayer was also significantly decreased after the use of the two inhibitors (*Figure 1E*). These data, combined with the morphological evidence, identify that the transmitter vesicles in VCs can mediate the chemical transmission of electrical excitation.

### Acetylcholine is the main transmitter component of the transmitter vesicles in ventricular cardiomyocytes

**Figure 2:**
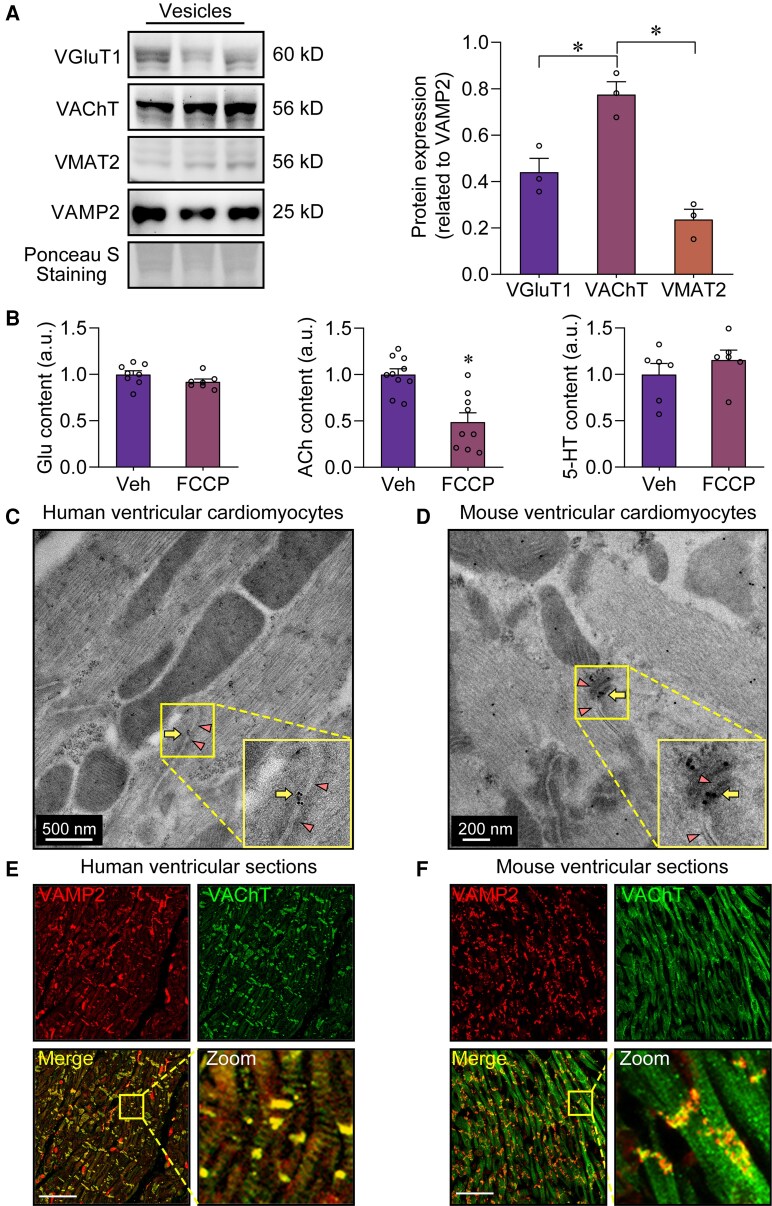
Detection of acetylcholine in transmitter vesicles from mouse ventricular cardiomyocytes. (*A*) Left, western blot confirmed that the vesicles within mouse ventricular cardiomyocytes expressed the vesicular acetylcholine transporters. Right, the bar graph summarizes the expression levels of vesicular transmitter transporters. *P < .05, calculated by one-way ANOVA; *n* = 3 per group. (*B*) Effect of the proton ionophore FCCP on the transmitter content in the extracted transmitter vesicles. *P < .05, calculated by Student’s *t*-test; *n* = 6–10 per group. (*C*, *D*) Immunogold electron microscopy demonstrated vesicle-like structures (arrows) with vesicular acetylcholine transporter immunoreactivity in ventricular cardiomyocytes from both humans and mice. The triangles indicate gap junctions. The diameter of each gold particle was 12 nm. (*E*, *F*) Immunofluorescence staining of vesicle-associated membrane protein 2 and vesicular acetylcholine transporter in human and mouse ventricular myocardial sections. Scale bar, 50 μm. Veh, vehicle

As shown in the above experiments, the electrical conduction velocity of the ventricles was significantly decreased following the inhibition of transmitter vesicle fusion, which suggests that these vesicles may contain excitatory transmitters (i.e. neurotransmitters). Thus, this study focused on the identification of excitatory transmitters. In the nervous system, excitatory transmitters mainly include glutamate (Glu), 5-hydroxytryptamine (5-HT), and ACh.^14,15^ In order to identify the types of transmitters contained in these transmitter vesicles, we first detected the presence of the transmitter vesicles in the extract by the transmitter vesicle markers and then identified the corresponding transmitters by the transmitter markers. Using size exclusion columns, we firstly extracted the transmitter vesicles from the isolated mouse VCs. Western blot consistently showed positivity for vesicle-associated membrane protein 2 (VAMP2), indicating that the extracted vesicles were transmitter vesicles (*Figure 2A*). Next, we identified the transmitter contained within the transmitter vesicles by using transmitter markers, i.e. vesicular transmitter transporters. The vesicular transmitter transporters of Glu, 5-HT, and ACh are vesicular glutamate transporter, vesicular monoamine transporter 2, and vesicular ACh transporter (VAChT), respectively. As shown in *Figure 2A*, the expression of VAChT in the transmitter vesicles extracted from the VCs was exclusively detected, while the other two kinds of vesicular transmitter transporters were barely detected, demonstrating that the transmitter in the vesicles from the VCs was mainly ACh.

Subsequently, we tried to provide further supporting evidence that the transmitter is indeed ACh by using biochemistry experiments. Usually, neurotransmitters are typically stored in neurotransmitter vesicles with an acidic environment inside, while destruction of the acidic environment is not conducive to the storage of neurotransmitters in vesicles.^16,17^ Thus, we used the proton ionophore carbonyl cyanide *p*-trifluoromethoxyphenylhydrazone (FCCP) to neutralize the acidic environment of the vesicles. It was found that FCCP intervention significantly reduced the ACh levels in the extracted transmitter vesicles, but did not significantly change the levels of Glu and 5-HT (*Figure 2B*). These data suggest that ACh is the primary candidate transmitter involved in the transmitter vesicles from the VCs.

Additionally, using the ACh vesicle marker, VAChT, we further studied the distribution of ACh vesicles in the VCs. Immunoelectron microscopy showed that ACh vesicles were primarily located near the gap junctions between the VCs in both humans and mice (*Figure 2C* and *D*). Immunofluorescence studies showed that the ACh vesicles located under the surface membrane (especially at both ends) of the VCs, co-localized with a classical marker of transmitter vesicles (VAMP2) (*Figure 2E* and *F*).

### Ventricular cardiomyocytes possess an intact endogenous cholinergic system

**Figure 3:**
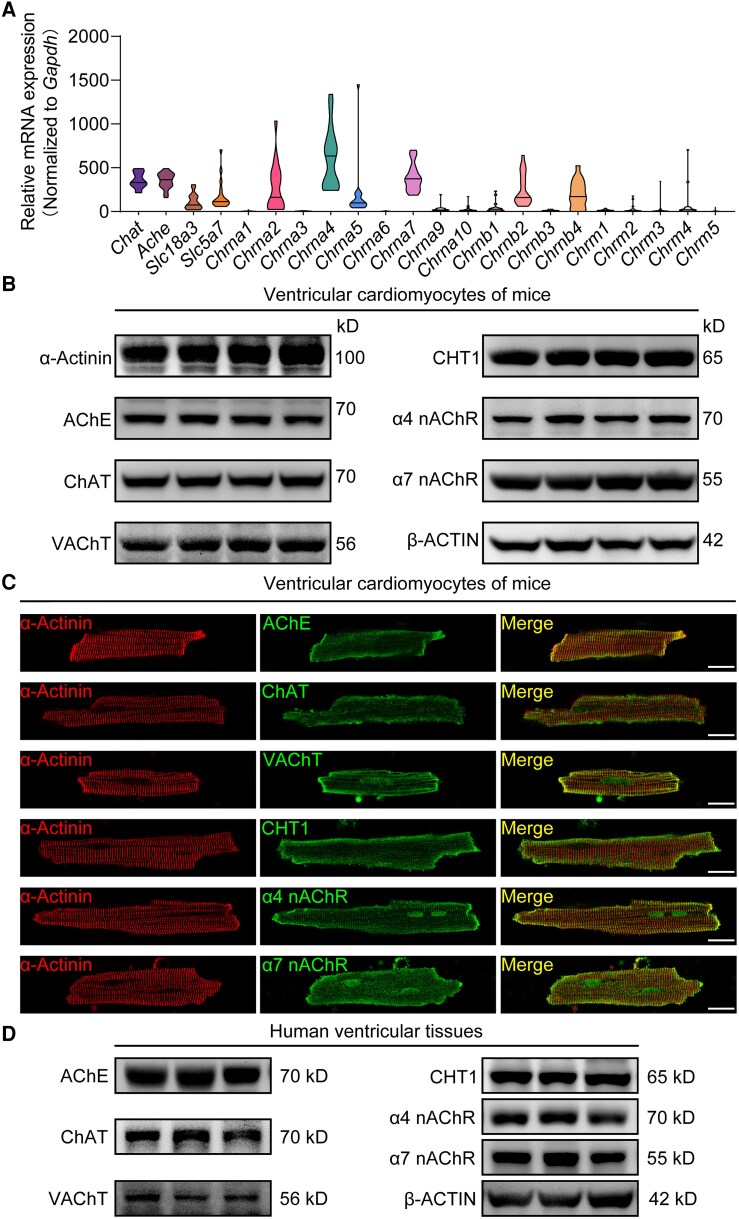
Ventricular cardiomyocytes express the core elements of the endogenous cholinergic system. (*A*) Violin plot showing the mRNA expression of the main genes of the endogenous cholinergic system in single ventricular cardiomyocytes. *n* = 17. (*B*) Representative western blot bands of the main elements of the endogenous cholinergic system in isolated ventricular cardiomyocytes from mice. (*C*) Immunofluorescence localization of acetylcholinesterase, choline acetyltransferase, choline transporter 1, vesicular acetylcholine transporter, and α4 and α7 nicotinic acetylcholine receptors in ventricular cardiomyocytes from mice. Cardiomyocytes were labelled with α-actinin. Scale bar, 20 μm. (*D*) Representative western blot bands of the main elements of the endogenous cholinergic system in human ventricles

The function of ACh as a transmitter depends on the existence of an endogenous cholinergic system.^15,18^ Thus, we isolated VCs from mouse ventricles to identify the core elements of the endogenous cholinergic system by single-cell quantitative polymerase chain reaction. As shown in *Figure 3A*, our data revealed that *Chat* (encoding choline acetyltransferase, ChAT), *Ache* (encoding acetylcholinesterase, AChE), *Slc18a3* (encoding VAChT), *Slc5a7* (encoding choline transporter 1, CHT1), and specific subtypes of nicotinic ACh receptors (nAChRs) were highly expressed in VCs. Subsequently, western blot further confirmed the presence of these endogenous cholinergic system components in the isolated mouse VCs (*Figure 3B*). In addition, immunofluorescence staining also validated the expression of ChAT, AChE, VAChT, CHT1, α4 nAChR, and α7 nAChR in isolated VCs and ventricular myocardial sections from mice (*Figure 3C* and Supplementary data online, *Figure S1*). Remarkably, we found that most of the components of the system tended to be located at the junctions at both ends of the VCs, demonstrating a potential spatial advantage in conducting the electrical excitation between VCs. Using similar techniques, we demonstrated that the core elements of the endogenous cholinergic system are also present in human VCs, and the distribution of this system in human VCs resembles that in mouse VCs (*Figure 3D* and Supplementary data online, *Figure S2*).

Moreover, we found that electrical pacing increased the ACh level in the culture medium containing isolated VCs. This increase can be significantly influenced by inhibiting ACh synthetase or ACh vesicle transport, suggesting that VCs can directly release ACh through a transmitter vesicle-dependent mechanism concurrent with VC excitation (see Supplementary data online, *Figure S3*). Taken together, these data confirm the presence of a complete cholinergic system in both human and mouse VCs.

### Acetylcholine puffs evoke instant and synchronous inward currents in ventricular cardiomyocytes in both a concentration- and a voltage-dependent manner

**Figure 4:**
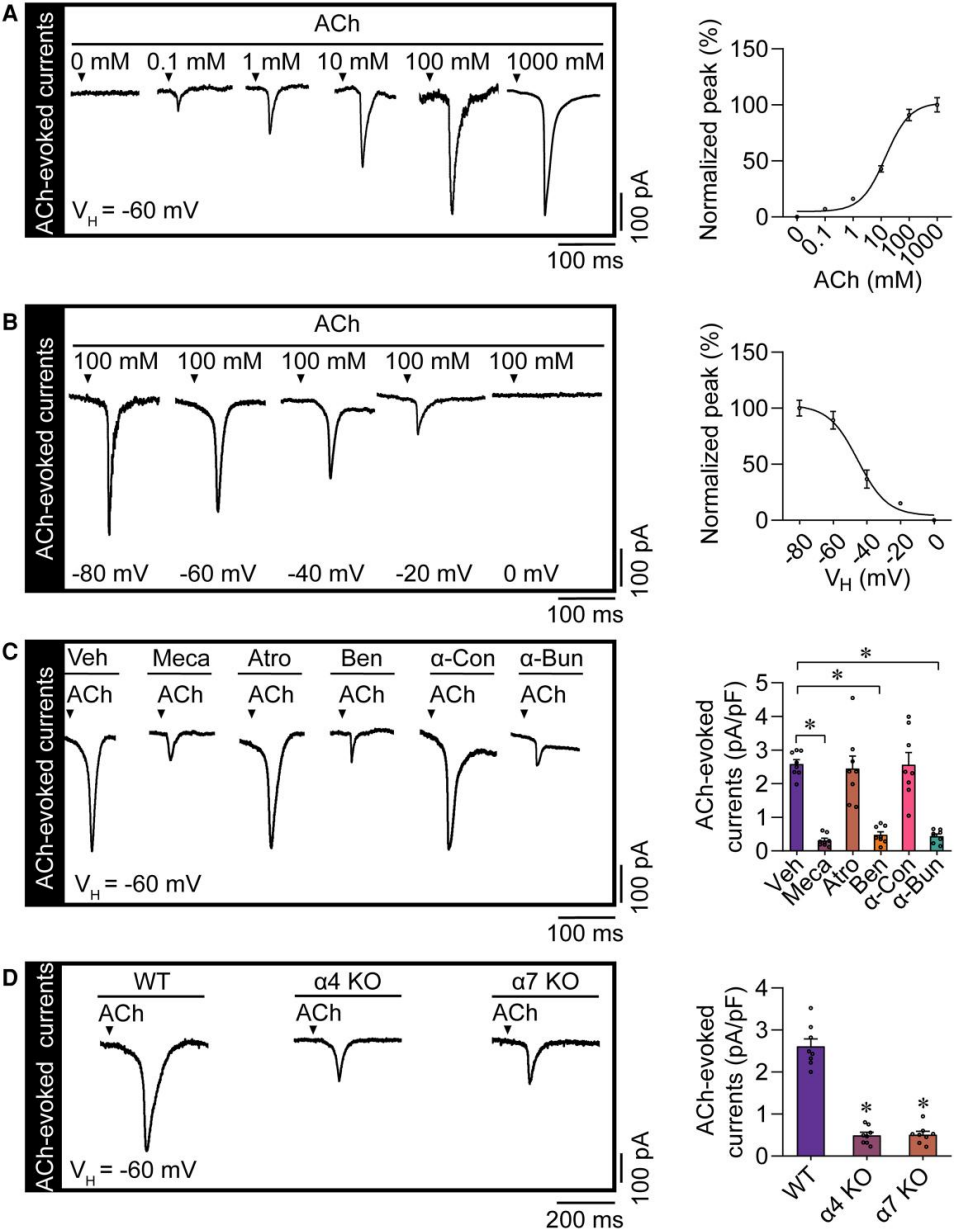
Identification of acetylcholine-evoked currents in mouse ventricular cardiomyocytes. (*A*) Representative patch-clamp recordings and the statistical analysis of the whole-cell currents elicited by different concentrations of acetylcholine (ACh) in mouse ventricular cardiomyocytes. The holding potential was set at −60 mV. *n* = 8–10 per group. (*B*) Representative patch-clamp recordings and the statistical analysis of the whole-cell currents elicited by 100 mM ACh in mouse ventricular cardiomyocytes. The holding potential was set at various voltages ranging from −80 to 0 mV. *n* = 8 per group. (*C*) The application of mecamylamine (Meca), benzethonium chloride (Ben), or α-bungarotoxin (α-Bun) can effectively decrease the acetylcholine-evoked inward currents. The holding potential was set at −60 mV. *P < .05 vs. vehicle, calculated by one-way ANOVA; *n* = 8 per group. (*D*) Representative current recordings in response to the ACh puff in ventricular cardiomyocytes isolated from wild-type mice and from α4 nicotinic ACh receptor knockout (α4 KO) and α7 nicotinic ACh receptor knockout (α7 KO) mice. The bar graph on the right provides a statistical summary of the ACh-evoked inward currents (right, *P < .05 vs. wild type, calculated by one-way ANOVA; *n* = 8 per group). WT, wild-type; Veh, vehicle; α-Con, α-conotoxin AuIB TFA; Atro, atropine; V_H_, holding potential

For a transmitter to perform its electrophysiological function in the nervous system, it usually first triggers the opening of selective ligand-gated channels on the postsynaptic membrane, which evokes an inward current in the postsynaptic membrane cell and leads to cell depolarization.^4,19^ As the release of transmitters in the nervous system is intermittent and not continuous, we investigated whether an ACh puff could elicit an inward current in VCs. The adult VCs were first isolated using enzymatic digestion, and the patch-clamp experiment was conducted. We initially set the holding potential of these isolated VCs to the resting potential of −60 mV, and the whole-cell currents were elicited by different concentrations of ACh puffs (100 psi, 30 ms) (*n* =8–10 for each group). As shown in *Figure 4A*, a transient inward current was instantly and synchronously evoked by spraying ACh (100 mM) on the surfaces of the cells. Importantly, we found a significant correlation between the current amplitude and the ACh concentration. Additionally, the peak currents induced by different concentrations of ACh puffs displayed a typical S-shaped curve response pattern found in muscles and neurons. In contrast, the Tyrode solution, as a control, did not evoke any currents (*Figure 4A*).

We also measured the inward currents evoked by the 100 mM ACh puff under different holding potentials ranging from −80 to +0 mV. The ACh-evoked currents were plotted against the different voltages, illustrating that ACh-evoked currents also exhibit voltage dependency, similar to neuronal responses to ACh (*Figure 4B*). This finding reflects the conserved and ubiquitous nature of ACh signalling across different types of excitable cells. In brief, we found that an ACh puff can evoke an inward current in VCs, suggesting that ACh can directly control the electrical activity of VCs like that of neurotransmitters in neurons.

### Acetylcholine puffs mediate the inward currents of ventricular cardiomyocytes through nicotinic acetylcholine receptors rather than muscarinic acetylcholine receptors

Typically, ACh exerts its effects through two main receptor types: nAChRs and muscarinic ACh receptors (mAChRs). Thus, we used specific inhibitors of nAChRs and mAChRs to determine the subtypes of the receptors mediating the ACh-evoked inward current in VCs. Firstly, we conducted a preliminary identification by using the non-selective inhibitors for nAChR or mAChR. We found that mecamylamine (the nAChR inhibitor) significantly inhibited the ACh-evoked inward current, while atropine (the mAChR inhibitor) exhibited no discernible effect on this current (*Figure 4C*). Subsequently, we used different specific nAChR inhibitors to further identify the core subtypes of the nAChRs in conducting the ACh-evoked inward currents in VCs. Among these inhibitors, α-bungarotoxin and benzethonium chloride (selectively inhibiting α4 and α7 nAChRs), but not the α-conotoxin AuIB TFA (selectively inhibiting α3 nAChR), can effectively decrease the ACh-evoked inward current in the VCs, implicating that α4 and α7 nAChR are key subtypes involved in the ACh-evoked inward current in VCs (*Figure 4C*).

In order to verify the gene function and rule out the off-target effect of the inhibitor used above, we utilized the CRISPR/Cas9 system to generate α4 and α7 nAChR knockout mice to characterize the role of the endogenous cholinergic system in cardiac electrical conduction (see Supplementary data online, *Figure S4*). Analyses of the cardiac function revealed no significant difference between α4 or α7 nAChR knockout mice and wild-type mice (see Supplementary data online, *Figure S4C* and *F*). We assessed whether an ACh puff could still evoke inward currents in VCs from α4 or α7 nAChR knockout mice. As shown in *Figure 4D*, application of ACh puffs successfully evoked the inward currents in the VCs from the wild-type mice, but this effect was markedly reduced in VCs from the α4 or α7 nAChR knockout mice. These data collectively suggest that the inward current evoked by ACh in VCs is mediated mainly through α4 and α7 nAChRs.

### Acetylcholine puffs significantly increase the excitability of ventricular cardiomyocytes by activating α4 and α7 nicotinic acetylcholine receptors

**Figure 5:**
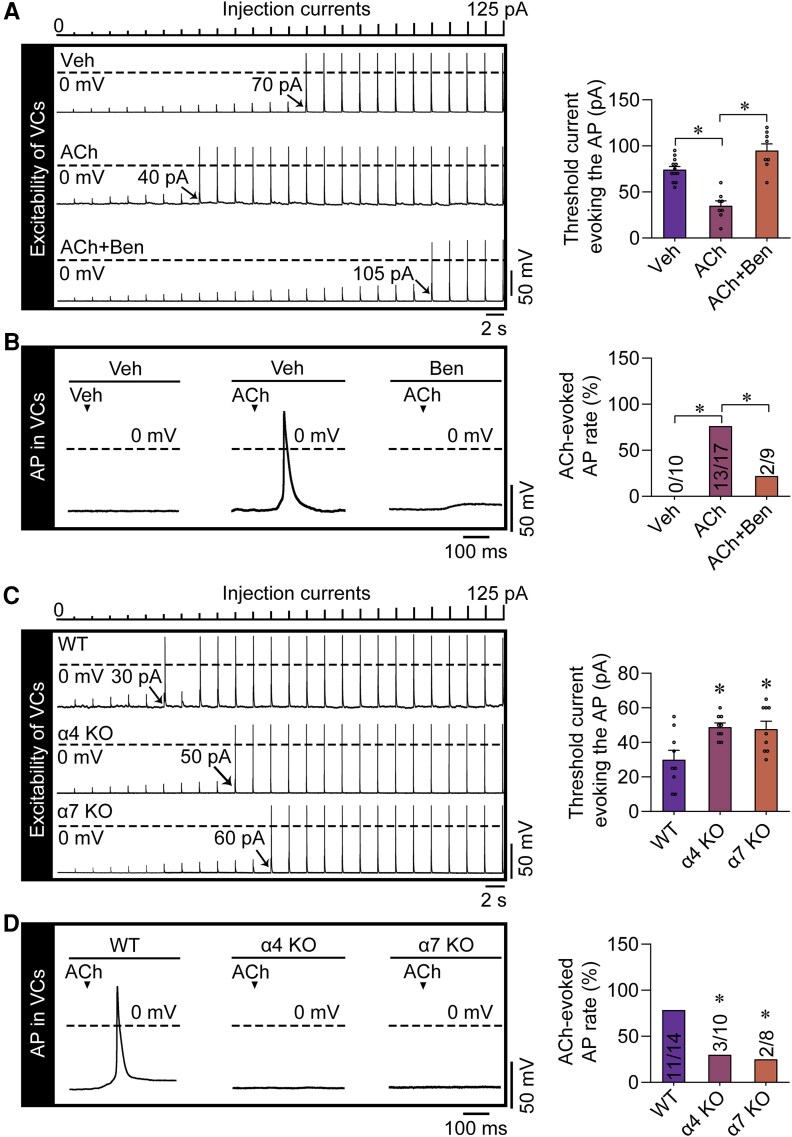
The endogenous cholinergic system regulates the excitability of mouse ventricular cardiomyocytes. (*A*) Action potentials were evoked by increasing injection currents from 0 to 125 pA by 5 pA step. The vehicle or 100 mM acetylcholine (ACh) puff was synchronously applied with the injection currents. The black arrows indicate the minimum threshold current evoking the action potential. The cells were pre-treated with vehicle (Veh) or 10 μM benzethonium chloride (Ben). Pooled data of the threshold currents required to evoke the action potential are presented at the right. *P < .05, calculated by one-way ANOVA; *n* = 8–12 per group. (*B*) Representative patch-clamp recordings and the statistical analysis of the action potential-evoking rates induced by the Veh or the 100 mM ACh puff in mouse ventricular cardiomyocytes. The cells were pre-treated with Veh or 10 μM Ben. The holding potential was set at −60 mV. Pooled data of the ACh-evoked action potential rates are presented at the right. *P < .05, calculated by Fisher’s exact test; *n* = 9–17 per group. (*C*) Action potentials were evoked in ventricular cardiomyocytes isolated from wild-type (WT), α4 nicotinic ACh receptor knockout (α4 KO), and α7 nicotinic ACh receptor knockout (α7 KO) mice by increasing injection current from 0 to 125 pA by 5 pA step. The 100 mM ACh puff was synchronously applied with the injection currents. The arrows indicate the minimum threshold current evoking the action potential. Pooled data of the threshold current required to evoke action potentials are presented on the right. *P < .05 vs. WT, calculated by one-way ANOVA; *n* = 9 per group. (*D*) Representative patch-clamp recordings and the statistical analysis of the action potential-evoking rates induced in ventricular cardiomyocytes isolated from WT mice and from α4 KO and α7 KO mice by the 100 mM ACh puff in mouse ventricular cardiomyocytes. The holding potential was set at −60 mV. Pooled data of the ACh-evoked action potential rates are presented at the right. *P < .05, calculated by Fisher’s exact test; *n* = 8–14 per group

As an ACh puff can cause inward currents in VCs, it is possible that ACh alters VC excitability. To prove this effect, we examined the minimum threshold current required for an AP to occur, which can reflect the excitation susceptibility. We combined the synchronous ACh puff stimulation with progressively increasing injection currents (ranging from 0 to 125 pA by 5 pA step) to measure the excitability of the isolated mouse VCs. The results showed that the ACh puffs notably reduced the minimum threshold current evoking Aps, thereby increasing the excitability of these cells (*Figure 5A*). Subsequently, we investigated whether ACh puffs could trigger APs in isolated mouse VCs by generating large inward currents. Unexpectedly, we found that high concentrations of ACh puffs were indeed capable of simultaneously inducing APs in these cells (*Figure 5B*). Our data also showed that benzethonium chloride, without changing the AP amplitude, not only reduced the minimum threshold current required to trigger AP generation but also inhibited ACh puff-induced AP generation in isolated VCs. This suggests that α4 or α7 nAChRs serve as the primary nAChR subunits regulating the excitability of VCs (*Figure 5A* and *B*). Compared with those from the wild-type mice, VCs from the α4 or α7 nAChR knockout mice displayed a higher threshold for the inward current to evoke APs and decreased ACh-evoked AP rates (*Figure 5C* and *D*), providing further evidence for the above data.

### The key components of the endogenous cholinergic system effectively control the conductivity of ventricular cardiomyocytes in the absence of foreign innervation

**Figure 6:**
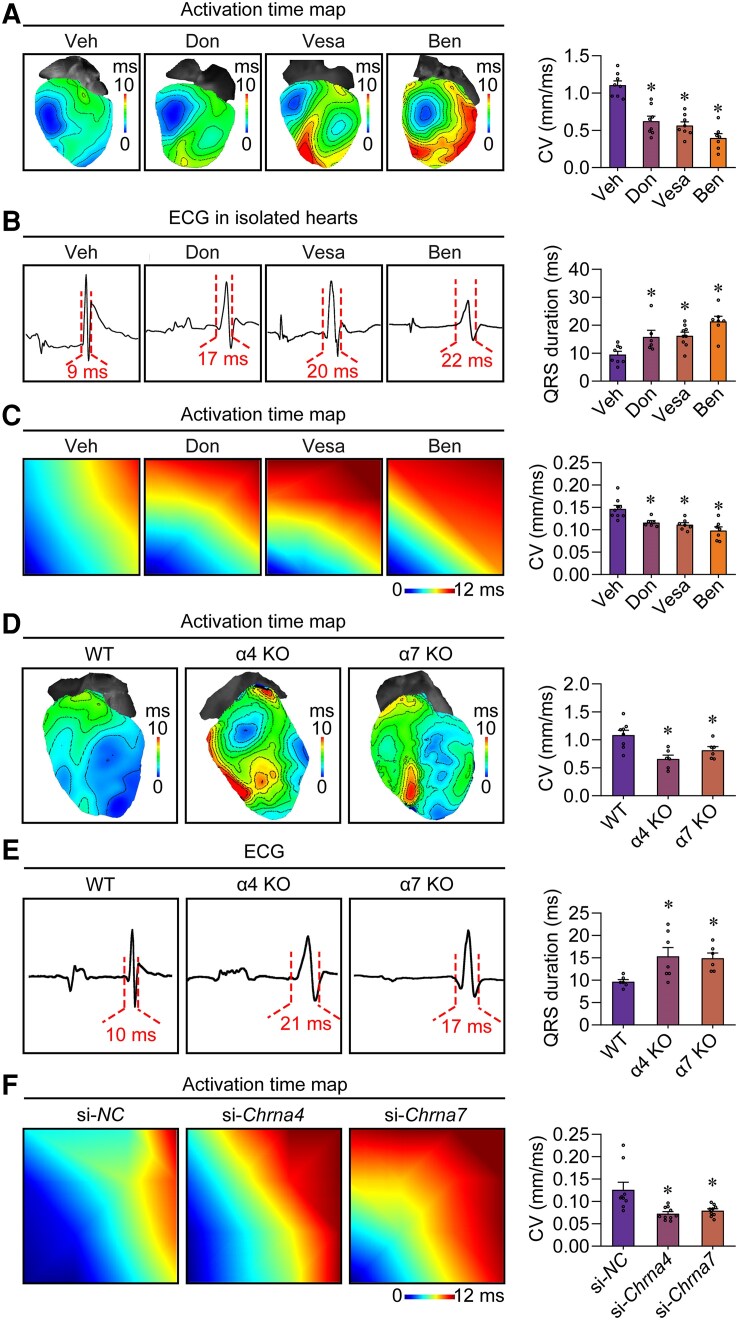
The endogenous cholinergic system regulates the conduction of electrical excitation in the ventricle. (*A*) Representative activation maps of isolated mouse hearts perfused with vehicle (Veh) or 10 μM inhibitors targeting the endogenous cholinergic system. Quantification of ventricular conduction velocity is shown in the bar graph (right, *P < .05 vs. Veh, calculated by one-way ANOVA; *n* = 7–8 per group). Donepezil (Don), an acetylcholinesterase inhibitor; vesamicol hydrochloride (Vesa), a vesicular acetylcholine transporter inhibitor; benzethonium chloride (Ben), an α4 and α7 nicotinic acetylcholine receptor inhibitor. (*B*) Representative electrocardiogram recordings of isolated mouse hearts perfused with vehicle or 10 μM inhibitors targeting the endogenous cholinergic system. A statistical summary of the QRS duration is shown in the bar graph (right, *P < .05 vs. vehicle, calculated by one-way ANOVA; *n* = 6–9 per group). (*C*) Representative images of activation time maps from multi-electrode array recordings and the statistical analysis of the conduction velocity (CV) in the monolayer of human induced pluripotent stem cell-derived cardiomyocytes (right, *P < .05 vs. vehicle, calculated by one-way ANOVA; *n* = 6–9 per group). (*D*) Representative activation time maps of hearts isolated from wild-type (WT), α4 nicotinic acetylcholine receptor knockout (α4 KO) and α7 nicotinic acetylcholine receptor knockout (α7 KO) mice under basal conditions (left). Quantification of the conduction velocity is shown in the bar graph (right, *P < .05 vs. wild-type, calculated by one-way ANOVA; *n* = 6–8 per group). (*E*) Representative *in vitro* electrocardiogram images of WT, α4 KO and α7 KO mice (left). A statistical summary of the QRS duration is shown in the bar graph (right, *P < .05 vs. wild-type, calculated by one-way ANOVA; *n* = 6–7 per group). (*F*) Representative images of the activation time maps from the multi-electrode array recordings and the statistical analysis of the conduction velocity in α4 or α7 nicotinic acetylcholine receptor-deficient human induced pluripotent stem cell-derived cardiomyocyte monolayer (right, *P < .05 vs. si-*NC*, calculated by one-way ANOVA; *n* = 9–10 per group)

Without the electrical excitation of cells, there would be no electrical conduction between cells.^20^ Therefore, electrical excitation of cells is the basis of electrical conduction between cells. Given that the ACh puff has a significant effect on the excitatory plasticity of VCs through the nAChR-mediated current, we hypothesized that the ACh may also alter the conductivity of ventricular electrical excitation. To exclude the influence of foreign innervation, we used *in vitro* experiments to verify the role of this endogenous cholinergic system in the conductivity of ventricular electrical excitation. Using optical mapping, we observed the effects of cholinergic system on ventricular conduction in denervated Langendorff-perfused hearts treated with different inhibitors targeting the key elements of the cholinergic system (donepezil as an AChE inhibitor, vesamicol hydrochloride as a VAChT inhibitor, and benzethonium chloride as an α4 and α7 nAChR inhibitor). As shown in *Figure 6A*, compared with the vehicle, all the inhibitors significantly reduced the ventricular conduction velocity, and the strongest inhibitory effect was achieved with benzethonium chloride. Consistent with this finding, the perfusion of these inhibitors also caused a progressive prolongation of the QRS duration (*Figure 6B*). To exclude confounding effects of neurohumoral factors on VCs, we also assessed their effects in the hiPS-CM monolayer cultured on the probe of multi-electrode array. The results confirmed that all these inhibitors slowed the conduction velocity in the hiPS-CM monolayer, with benzethonium chloride achieving the greatest inhibition of electrical conduction (*Figure 6C*). Moreover, the conduction velocity of ventricular electrical excitation was significantly decreased in the α4 or α7 nAChR-deficient hearts (*Figure 6D*). Electrocardiogram recording revealed a widened QRS duration (representing the electrical excitation conduction of the ventricles) in the Langendorff-perfused hearts from the α4 or α7 nAChR knockout mice (*Figure 6E*). It is important to note that the Langendorff-perfused hearts exhibited intermittent dropped QRS complex (loss of QRS complex after P wave) as the frequency of externally applied electrical stimulation gradually increased (see Supplementary data online, *Figure S6*). We found no statistically significant difference in P wave duration (representing the atrial conduction), PR interval (the time from the onset of P wave to the start of QRS complex, representing the conduction of electrical excitation between the atria and ventricles), heart rate, and heart rate variability between α4 or α7 nAChR knockout mice and wild-type mice (see Supplementary data online, *Table S1*). We also found that α4 or α7 nAChR deficiency significantly slowed the conduction velocity in the hiPS-CM monolayer (*Figure 6F* and Supplementary data online, *Figure S5*). To further exclude the influence of nAChR in non-cardiomyocytes, we used an AAV9 virus with a cardiomyocyte-specific promoter TNNT2 (cardiac troponin T) to selectively knockdown the *Chrna4* or *Chrna7* in cardiomyocytes (see Supplementary data online, *Figure S7*). By using the optical mapping and ECG recording, we observed that the cardiomyocyte-specific *Chrna4* or *Chrna7* deficiency significantly slowed the ventricular conduction velocity and prolonged the QRS duration compared with the control group (see Supplementary data online, *Figure S8*). These results suggest that the cholinergic system regulates both excitability and conductivity of VCs.

Previous studies reported that blocking or damaging the gap junction can inhibit but not completely terminate electrical conduction.^21,22^ Thus, we hypothesized that the conduction of electrical impulses between VCs is shared by gap junctions and this endogenous cholinergic system. To distinguish the effects of these two systems, we inhibited the gap junction and endogenous cholinergic system separately or simultaneously in the Langendorff-perfused hearts. We found that heptanol, a gap junction uncoupler, or benzethonium chloride, an α4 and α7 nAChR inhibitor, slowed ventricular electrical conduction, whereas their combined use completely blocked conduction (see Supplementary data online, *Figure S9A*). Consistent with the above results acquired by optical mapping, ECG recording of Langendorff-perfused hearts also showed that the application of benzethonium chloride alone or the gap junction uncoupler alone could diminish the QRS amplitude and prolong the QRS duration, while the application of these two inhibitors together resulted in the complete disappearance of the QRS complexes (*n* = 5), indicating that the ventricular electrical conduction is completely interrupted (see Supplementary data online, *Figure S9B*). These findings suggest that the endogenous cholinergic system and gap junctions work together to regulate the conduction of electrical impulses in the ventricular myocardium.

Both VCs and Purkinje cells can affect electrical conduction in the ventricle.^23,24^ To exclude the involvement of Purkinje cells in the conduction of electrical excitation mediated by this endogenous cholinergic system, we isolated ventricles with Purkinje fibres from rabbits and determined the effect of benzethonium chloride on the electrical conduction velocity. We observed that benzethonium chloride can reduce ventricular conduction velocity, while Purkinje fibre conduction velocity was not affected. This suggests that the endogenous cholinergic system primarily affects electrical excitation conduction through VCs (see Supplementary data online, *Figure S10*).

### The defects of the endogenous cholinergic system are closely related to ventricular arrhythmia, and intervention targeting the system can effectively prevent and treat ventricular arrhythmia

**Figure 7:**
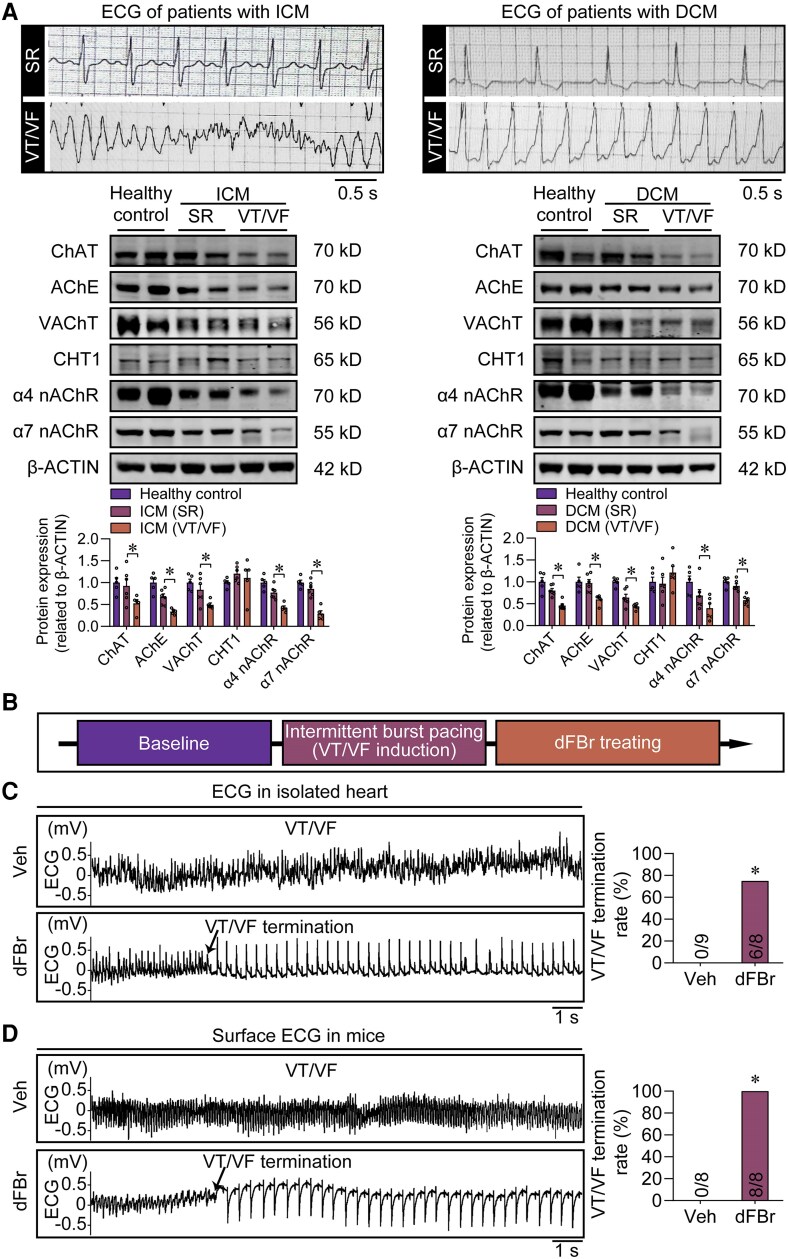
Defects in this endogenous cholinergic system are associated with fatal ventricular arrhythmias. (*A*) Top, representative electrocardiograms from patients diagnosed with ischaemic cardiomyopathy or dilated cardiomyopathy. Middle, representative western blot images showing different expression levels of key members of the endogenous cholinergic system in the ventricular myocardium from healthy control and ischaemic cardiomyopathy (ICM) or dilated cardiomyopathy (DCM) patients with or without a history of ventricular tachycardia/ventricular fibrillation (VT/VF) episodes. Bottom, the bar graph summarizes the expression levels of the key members of the endogenous cholinergic system. *P < .05, calculated by Student’s *t*-test; *n* = 5–6 per group. Sinus rhythm, SR. (*B*) Experimental design for analysing the effects of the desformylflustrabromine (dFBr) on treating the ventricular arrhythmias. (*C*) Representative *in vitro* electrocardiogram recordings showed that VT/VF can be terminated by the application of the dFBr but not the vehicle (Veh). The bar graph shows the VT/VF termination rate in the two groups (right). *P < .05, calculated by Fisher’s exact test, *n* = 8–9 per group. (*D*) Representative *in vivo* electrocardiogram recordings showed that VT/VF can be terminated by the application of dFBr but not the Veh. The bar graph shows the VT/VF rate in the two groups (right). *P < .05, calculated by Fisher’s exact test, *n* = 8 per group

Ventricular arrhythmias, often associated with multiple underlying pathologies, such as ischaemia and heart failure, are a major clinical challenge.^25^ To investigate the association between the endogenous cholinergic system and ventricular arrhythmia, we examined the alteration of the endogenous cholinergic system in ventricular myocardium from healthy donors and patients diagnosed with either ischaemic cardiomyopathy (ICM) or dilated cardiomyopathy (DCM), with a focus on those with and without ventricular arrhythmias. We investigated a cohort of 11 patients diagnosed with ICM and 12 patients diagnosed with DCM, all of whom underwent heart transplantation. There were no significant differences in age or sex observed among the four groups (see Supplementary data online, *Table S2*). We examined the routine ECG and Holter ECG data of these patients and measured the levels of the endogenous cholinergic system in their ventricular myocardium. We divided the enrolled heart failure patients into four groups: the ventricular tachycardia/ventricular fibrillation (VT/VF) ICM patient group, the sinus rhythm ICM patient group, the VT/VF DCM patient group, and the sinus rhythm DCM patient group. Consistent with the previous animal data, we found that the expression levels of ChAT, CHT1, AChE, VAChT, and α4 and α7 nAChR were significantly lower in the VT/VF patient group than those in the sinus rhythm patient group, regardless of whether the underlying cause was ICM or DCM (*Figure 7A*).

To further substantiate the association between the endogenous cholinergic system and ventricular arrhythmia, we performed verification experiments in two animal models. Myocardial infarction (MI)-induced and transverse aortic constriction (TAC)-induced heart failure mouse models were created and subjected to programmed electrical stimulation to induce VT/VF. Following this procedure, the mice were divided into two groups: the VT/VF group and the sinus rhythm group. Western blot revealed that, in the VT/VF group, the expression levels of key components in the endogenous cholinergic system were significantly lower than those in the sinus rhythm group, regardless of whether the mice were from the MI-induced or TAC-induced heart failure mouse model (see Supplementary data online, *Figure S11*). Furthermore, we assessed the susceptibility to arrhythmia in our α4 nAChR and α7 nAChR knockout mice. By using a programmed stimulation-induced VT/VF model, we observed that either α4 or α7 nAChR deficiency led to an increased incidence of arrhythmic events (see Supplementary data online, *Figure S12*). These data highlight the important role of the α4 and α7 nAChR in mediating the excitability and conductivity of VCs. Collectively, both the experimental animal and human arrhythmia data suggest that endogenous cholinergic defects link to fatal cardiac arrhythmias.

As down-regulation of the endogenous cholinergic system is associated with susceptibility to fatal ventricular arrhythmias in both patients and animal models, the endogenous cholinergic system could become a target for intervention in preventing or treating fatal VT/VF. To test this hypothesis, we investigated the target role of the key component in the endogenous cholinergic system (such as some specific nAChRs) in preventing or treating the obtained VT/VF in the mouse models. We chose a positive allosteric modulator of the α4 nAChR [desformylflustrabromine (dFBr)], which can directly bind the α4 nAChR and amplify the ACh-induced responses, to detect whether the agonist could prevent or treat VT/VF in the mouse models. The result showed that the dFBr not only significantly reduced the VT/VF induction rates but also effectively terminated the ongoing VT/VF *in vitro* and *in vivo* (*Figure 7B–D* and Supplementary data online, *Figure S13*). These results support a strategy targeting the α4 nAChR as a promising way to prevent fatal ventricular arrhythmias in clinical practice.

## Discussion

The heart is innervated by the parasympathetic nervous system, which utilizes its cholinergic system to regulate heart rhythm.^26^ As a transmitter released by the parasympathetic nerve, ACh significantly influences the cardiac conduction system. It decreases the activity of pacemaker cells in the sinoatrial node, leading to a reduction in the pacing frequency of the sinoatrial node. In extreme scenarios, ACh can cause sinus bradycardia, sinoatrial block, and sinus arrest and result in cardiogenic syncope.^27,28^ Furthermore, ACh not only reduces the pacing frequency of the atrioventricular node but also diminishes its conduction activity, extends the atrioventricular conduction time, and may lead to partial or complete atrioventricular block.^29,30^ Acetylcholine also impacts the atrial myocardium, though it has not been shown to have a significant effect on the ventricular myocardium.^31^ This study reveals that ACh released from vesicles in VCs can act as a transmitter in the heart's endogenous cholinergic system, activating nAChRs on the surface membrane of VCs, thereby controlling the electrical conduction in the heart.

The conduction of electrical impulses in the heart is undertaken by the cardiac conduction system and cardiomyocytes. The cardiac conduction system consists of the sinoatrial node, the atrioventricular node, the His bundle, and the Purkinje fibres.^32^ Cardiomyocytes mainly include atrial cardiomyocytes and VCs. Traditionally, it is believed that within both the cardiac conduction system and VCs, the pathway for electrical impulses is through gap junction channel. Transmitter-mediated electrical conduction in the heart has seldom been reported. We recently reported that atrial cardiomyocytes possess an endogenous glutamatergic system that facilitates electrical conduction between atrial cardiomyocytes.^9^ The identification of the glutamatergic system of atrial cardiomyocytes, together with the subsequent discovery of the cholinergic system of the VCs presented in this study, unveils promising new avenues for research into heart rhythm regulation. The development of the concepts of synapses, synaptic receptors, chemical receptors, chemical transmission, and transmitters of electrical excitation was a major advance in the field of neurobiology. However, these concepts have little to do with the heart. Our findings identify a new member of the electrical conduction family in the heart. Whether the sinoatrial node and atrioventricular node also possess unique transmitter systems represents a scientifically intriguing question that merits further investigation.

Abnormal cardiac conduction triggers the onset of multiple arrhythmias, including fatal ventricular arrhythmias and sudden cardiac death.^24,33,34^ Our findings, viewed from the perspective of chemical transmission of electrical excitation, suggest a novel potential mechanism for aberrant electrical conduction under pathological conditions. The confirmation of a link between this endogenous cholinergic system and fatal ventricular arrhythmias in patients highlights its significance in predicting the onset of arrhythmia. Interestingly, the expression of specific elements of the endogenous cholinergic system was changed in the ventricular myocardium of patients who experienced fatal ventricular arrhythmias. Similar results were also noted in experimental animal models, suggesting a strong correlation with fatal ventricular arrhythmias. Still, the findings need to be validated through larger clinical sample sizes and further intervention trials to establish their significance and applicability.

The discovery of the heart’s intrinsic cholinergic system is of great significance for understanding how smoking or nicotine addiction leads to arrhythmias. Smoking contributes to the occurrence and progression of cardiovascular disease through nicotine, carbon monoxide, oxidant gases, and other toxic chemicals, with nicotine being closely linked to arrhythmias and cardiovascular mortality.^35–37^ As VCs possess an intrinsic cholinergic system, it is hypothesized that nicotine from tobacco or e-cigarettes might directly affect the heart’s nAChRs, altering electrical conductivity within the ventricles, increasing the risk of ventricular arrhythmias, and potentially leading to fatal outcomes. Nicotine mainly exerts its effects on the central nervous system’s nAChRs but can also act through specific subtypes of nAChRs in the autonomic nervous system that regulate the heart.^38^ Undoubtedly, the role of cardiac nAChRs in smoking-related arrhythmias and cardiovascular events is a challenging scientific issue that requires further in-depth exploration. It should be noted that in our study, dFBr, a positive allosteric regulator of nAChR, effectively prevents and treats arrhythmia. In contrast, nicotine, an nAChR agonist, has been reported to induce arrhythmia in the literature.^37–39^ This discrepancy deserves further research. We hypothesize that the opposing effects may be due to differences in the dynamics of their interaction with nAChR.

The therapeutic effects of most antiarrhythmic drugs are not satisfactory, with some even inducing fatal arrhythmias or sudden cardiac death.^40^ However, to date, few fundamental breakthroughs have been made in the development of novel antiarrhythmic drugs. The primary challenges in arrhythmia treatment stem from the scarcity of innovative theories and targets. Currently, interventions for ventricular arrhythmias primarily focus on ion channels located on the surface membrane of VCs and gap junction channels facilitating communication between VCs. Considering the limited efficacy and arrhythmogenic side effects of current treatment, targeting the endogenous cholinergic system may present a potentially innovative alternative strategy, expanding beyond the classical approaches.

This study has three limitations. First, while we verified the presence of transmitter vesicles among VCs, we could not identify the typical chemical synaptic structure akin to those found in neuronal synapses. Second, given the complexity of the spatial structure, it is challenging to ascertain the respective contribution of gap junction-mediated electrical conduction and transmitter-mediated electrical conduction to overall ventricular electrical conduction in the ventricles. However, our experimental data showed that neither gap junction inhibitor alone nor an inhibitor of cholinergic system elements alone can completely block the electrical conduction of ventricular myocardium. Instead, the combination of the two can entirely block the electrical conduction in the ventricular myocardium. This indicates that both elements independently contribute to the electrical conduction process within the ventricular myocardium. Finally, the role of transmitters other than ACh in initiating electrical excitation in ventricular myocardium has not been thoroughly investigated, making a significant gap in the current understanding. However, our data showed that in addition to ACh vesicles, a small number of Glu vesicles and 5-HT vesicles are also present in VCs. The existence of complete glutamatergic systems and serotonergic systems within the heart, as well as their functions, remains to be further explored to ascertain their impact on cardiac electrophysiology.

In summary, our study uncovers that VCs possess an intact cholinergic system, which facilitates the conduction of electrical excitation among VCs via chemical transmission patterns akin to those observed in neurons, thereby initiating the contractile activity of the ventricles. Our findings indicate that abnormalities within the endogenous cholinergic system of VCs are linked to the occurrence of fatal ventricular arrhythmias in patients. We have identified a novel entity responsible for mediating the conduction of electrical excitation in the heart, thereby introducing a new theoretical prototype for the pathogenesis of cardiac arrhythmias and providing a series of potential targets for the intervention of cardiac arrhythmias (*Structured Graphical Abstract*).

## Supplementary Material

ehae699_Supplementary_Data

## Declarations

### Data Availability

All data are available in the main text or the Supplementary data.
