# Supplementary material for: An endogenous cholinergic system controls electrical conduction in the heart
Source: Eur Heart J. 2024 Oct 22;46(13):1232–46. doi: 10.1093/eurheartj/ehae699 (PMC11959186; doi:10.1093/eurheartj/ehae699)
Supplement: ehae699_Supplementary_Data [file ehae699_supplementary_data.zip › Supplementary materials.docx]

**Supplementary Materials For**

**An endogenous cholinergic system controls electrical conduction in the heart**

**Authors:**

Duanyang Xie^1,2,3,4†^, Ke Xiong^1,3†^, Nianguo Dong^5†^, Guanghua Wang^1,3,4†^, Qicheng Zou^1,3†^, Beihua Shao^1,3^, Zhiwen Chen^1,3^, Luxin Wang^3^, Yu Kong^6^, Xu Wang^6^, Xuling Su^3^, Wenli Bai^1,7^, Jian Yang^1,2,3^, Yi Liu^1,2,3^, Bin Zhou^8^ and Yi-Han Chen^1,2,3,4,9^*

**Affiliations:**

^1^State Key Laboratory of Cardiovascular Diseases, Shanghai East Hospital, School of Medicine, Tongji University, Shanghai 200120, China. ^2^Shanghai Arrhythmia Research Center, Shanghai East Hospital, School of Medicine, Tongji University, Shanghai 200120, China. ^3^Department of Cardiology, Shanghai East Hospital, School of Medicine, Tongji University, Shanghai 200120, China. ^4^Department of Pathology and Pathophysiology, School of Medicine, Tongji University, Shanghai 200092, China. ^5^Department of Cardiovascular Surgery, Union Hospital, Tongji Medical College, Huazhong University of Science and Technology, Wuhan 430022, China. ^6^Electron Microscopy Facilities of Center for Excellence in Brain Science and Technology, Chinese Academy of Science, Shanghai 200031, China. ^7^Jinzhou Medical University, Liaoning 121000, China. ^8^Center for Excellence in Molecular Cell Science, Chinese Academy of Sciences, Shanghai, China. ^9^Research Units of Origin and Regulation of Heart Rhythm, Chinese Academy of Medical Sciences, Shanghai 200092, China.

†These authors contributed equally: Duanyang Xie, Ke Xiong, Nianguo Dong, Guanghua Wang and Qicheng Zou. *Corresponding author. Email: yihanchen@tongji.edu.cn

**This file includes:**

Methods

Figures S1-S13

**Methods**

**Animals**

This study conforms to the requirements of the Guide for the Care and Use of Laboratory Animals established by the U.S. National Institutes of Health. All animal experiments were approved by the Animal Care and Use Committee of at Tongji University School of Medicine. The C57BL/6 mice aged 6-8 weeks were purchased from Shanghai Sippr-BK laboratory animal Co. Ltd (China). The New Zealand White rabbits aged 3-4 months were purchased from Shanghai JieSiJie Laboratory Animal Center (China).

**Transgenic mice**

*Chrna4* knockout mice and *Chrna7* knockout mice were generated by GemPharmatech Co., Ltd. (China). The mice were born following a normal Mendelian ratio, exhibited no discernible developmental defects, and were fertile with a normal litter size. All the mice were housed under a 12-hour light/dark cycle in a specific pathogen-free facility with *ad libitum* access to sterile water and food.

**Patients and sample**

Ventricular myocardial samples were collected from discarded human donor hearts and patients with end-stage heart failure who underwent heart transplantation at Wuhan Union Hospital, Wuhan, China. These patients were diagnosed with either ischemic cardiomyopathy (ICM) or dilated cardiomyopathy (DCM). Informed consent was obtained from all the enrolled subjects. The experimental protocol and sample collection adhered to the principles outlined in the Declaration of Helsinki and received approval from the ethics committee of the Wuhan Union Hospital, Wuhan, China.

**Transmission electron microscopy**

Fresh ventricular myocardium was sectioned into 1 mm tissue blocks and immediately immersed in ice-cold 2.5% glutaraldehyde (buffered with 0.1 M phosphate buffer, pH 7.4). The samples were fixed for at least 1 h at 4°C and then repeatedly washed in phosphate buffer and postfixed in 1% osmium tetroxide for 1 h. The fixed specimens were dehydrated in increasing concentrations of ethanol (10%, 50%, 70%, and 90%), embedded in 100% epoxy resin and left to polymerize at 55°C in 5% CO_2_ for 36 h. Next, the resin blocks were sectioned with an ultramicrotome, and the ultrathin sections were placed on the grids and stained with uranyl acetate and lead citrate solution for transmission electron microscopy image observation (JEOL, Japan).

**Immunoelectron microscopy**

For the analysis of VAChT localization at the ultrastructural level, fresh ventricular tissues were prepared for immunoelectron microscopy. Briefly, fresh ventricular tissues were sectioned into 1 mm tissue blocks, fixed in 2% paraformaldehyde and 0.5% glutaraldehyde (buffered with 0.1 M phosphate buffer, pH 7.4) for 90 min at room temperature, and immersed in 4% paraformaldehyde in the same buffer at 4°C overnight. The samples were subsequently washed with PBS, embedded in 12% gelatin, infiltrated in 2.3 M sucrose and frozen in liquid nitrogen. Ultrathin cryosections were obtained using a Leica EM FC7 cryo-ultramicrotome and collected on copper-formvar-carbon-coated grids. VAChT localization was revealed using anti-VAChT (Abcam, USA) and anti-rabbit IgG gold (gold size 12nm). Tissue sections were imaged at ×40000 magnification using a transmission electron microscope (JEOL, Japan).

**Optical mapping**

Optical mapping was conducted following established methods^1,2^. Briefly, adult mice or rabbits were anesthetized, and the chest was surgically opened. Subsequently, the lung, thymus and fat tissue were carefully dissected and removed, and the heart was promptly excised in ice-cold Tyrode’s solution containing (in mM) 140 NaCl, 5.4 KCl, 1.8 CaCl_2_, 1.2 KH_2_PO_4_, 5 HEPES, 1 MgCl_2_, and 5.5 glucose (pH adjusted to 7.4 with NaOH). After Langendorff perfusion at 37°C with oxygenated Tyrode’s solution, the heart was allowed to recover and then loaded with Di-4-ANEPPS (10 μM, AAT Bioquest, USA). To minimize motion artifacts, blebbistatin (MCE, USA) was added to the perfusate at a final concentration of 10 μM. Optical mapping was conducted on the whole heart or the ventricular endocardium. Images were captured using a high-speed camera (MiCAM ULTIMA, BrainVision, Inc., Japan) at 1,000 frames/second. Data collection and analysis were conducted using BV Workbench software (SciMedia, USA). The activation time was determined as the time point at which the maximum change in fluorescence occurred over time (dF/dt) for each fluorescent signal.

**Multi-electrode array (MEA) recording in human induced pluripotent stem cell-derived cardiomyocytes (hiPS-CM) monolayer**

The hiPS-CM were obtained from the HELP Stem CellInnovations (Nanjing, China). After recovery, the cells were cultured in a dish precoated with fibronectin (2 μg/μL, Sigma, USA) with cardiomyocyte recovery media (HELP Stem CellInnovations, China) at 37 °C, under 5% CO_2_. After 24 hours, cells were then dissociated and adjusted to a concentration of 4 x 10^6^ cells/mL. Cell suspensions (10 μL each) were seeded onto CytoView MEA 24 plates (Axion BioSystems, USA). The MEA setup was optimized to automatically regulate the environmental conditions (37°C and 5% CO_2_), ensuring stable temperature and pH for the medium. Data collection was facilitated using the AxIS Navigator software (Axion BioSystems, USA).

The siRNA-mediated knockdown of *Chrna4* and *Chrna7* was performed with the lipofectamine RNAiMAX (Invitrogen, USA) following the manufacturer’s guidelines. Briefly, the cultured hiPS-CM were transfected with a final concentration of ∼20 pmol siRNA together with 4.5 μL lipofectamine RNAiMAX per 500 μL. The data were collected after 72 h post-transfection. All the siRNA sequences are listed in Supplementary information, *Table S3*.

**Vesicle extraction for mouse ventricular cardiomyocytes (VCs) and enzyme-linked immunosorbent assays (ELISAs)**

For the isolation of vesicles from adult mouse VCs, cardiomyocytes were isolated from the ventricles as previously described, with minor modifications^3^. Mice (6-8 weeks) were injected with 100 μl of heparin (100 IU/mouse) before being anesthetized with pentobarbital (40 mg/kg). The hearts were removed and perfused at 37 °C with Ca^2+^-free buffer containing (in mM) 140 NaCl, 5.4 KCl, 1.2 KH_2_PO_4_, 5 HEPES, 1 MgCl_2_, and 5.5 glucose (pH adjusted to 7.4 with NaOH) until the effluents became clear. Next, enzymatic digestion was performed with collagenase type II (0.5 mg/mL, Gibco) for 10 min. When the hearts became slightly pale and flaccid, the ventricles were removed, cut into small pieces in stop buffer (Ca^2+^-free solution with 1 mg/mL bovine serum albumin), and filtered through a 100 µm cell strainer. The isolated VCs were counted, and the cells were centrifuged at 500 × g for 5 minutes for subsequent vesicle extraction. The vesicles within the VCs were extracted using a Cellular Vesicle Isolation Kit (BestBio, China) according to the manufacturer’s instructions. The purity and characteristics of the extracted vesicles were assessed by SDS‒PAGE and Western blot. The neurotransmitter levels were measured and quantitated using ELISA kits (Glutamate ELISA Kit, SinobestBio; ACh ELISA Kit, Elabscience Biotechnology, China; 5-HT ELISA Kit, Elabscience Biotechnology, China) according to the manufacturer’s instructions.

**Western blot**

Total protein was extracted through a conventional procedure by RIPA lysis buffer (Beyotime Biotechnology, China) with a protease inhibitor cocktail (Beyotime Biotechnology, China) at 4 °C. The protein concentrations were determined with a BCA kit (Beyotime Biotechnology, China), and equal amounts of protein were separated via 10% SDS‒PAGE (Thermo Fisher Scientific, USA) and subsequently transferred onto PVDF membranes (Millipore, USA). Next, the membranes were blocked with 5% nonfat milk in TBST (TBS, 0.1% Tween-20) buffer for 1 h at room temperature and then incubated with primary antibodies, including VAMP2 (Proteintech, USA), VGluT1 (Synaptic Systems, USA), VAChT (Abcam, USA), VMAT2 (Proteintech, USA), AChE (Proteintech, USA), ChAT (Invitrogen, USA), CHT1 (Proteintech, USA), α4 nAChR (Origene, USA), α7 nAChR (Proteintech, USA), β-ACTIN (Santa Cruz, USA) and GAPDH (Proteintech, USA) antibodies, overnight at 4°C. The next day, the PVDF membranes were incubated with a conjugated secondary antibody (Invitrogen, USA) for 1 h at room temperature, and the bands were visualized using a ChemiDoc Touch Gel Imaging System (Bio-Rad, USA).

**Immunofluorescence analysis**

For immunofluorescence staining of the ventricular sections, the samples were fixed with 4% paraformaldehyde, paraffin-embedded, and sectioned. Tissue sections were deparaffinized with xylene and rehydrated with a series of decreasing concentrations of ethanol. Antigen retrieval was performed by microwaving the sections slides in citrate solution for 10 min. The sections slides were then blocked with 10% goat serum and incubated with primary antibodies against VAMP2 (Proteintech, USA), VAChT (Abcam, USA), AChE (Proteintech, USA), ChAT (Invitrogen, USA), CHT1 (Proteintech, USA), α4 nAChR (Origene, USA) and α7 nAChR (Proteintech, USA) at 4 °C overnight. The next day, the sections were washed in PBS containing 0.1% Tween-20 (PBST) three times to remove unbound antibodies and then incubated with the appropriate fluorescence-conjugated secondary antibody (Invitrogen, USA) in PBST with 10% goat serum for 1 h at room temperature.

For the immunofluorescence analysis of mouse VCs, cells were fixed with fresh 4% paraformaldehyde for 13 min and washed twice with PBS for 5 min. The fixed cells were then permeabilized in 0.1% Triton-containing PBST for 10 min, blocked with 4% normal goat serum for 1 h at room temperature, and incubated with primary antibodies at 4 °C overnight. Next day, the cells were then washed with PBST and incubated with the appropriate fluorescence-conjugated secondary antibody (Invitrogen, USA) for 1 h at room temperature. After three washes with PBST, the stained cells were imaged by a Leica STELLARIS 5 confocal microscope (Leica, Germany).

**Quantitative real-time PCR (qPCR)**

For single-cell RNA extraction, individual cells were manually picked under the microscope and transferred into 2.5 μL cell lysis buffer to extract total RNA. RNA products were reversed transcribed with Maxima H Minus RT (Invitrogen, USA) and then amplificated for 18 PCR cycles with SeqAmp DNA Polymerase (Takara, Japan). cDNA quantification was performed with Qubit 4.0 (Thermo Fisher, USA). The cDNA was properly diluted and used as template for qPCR. All the qPCR reactions were performed with the Taq Pro Universal SYBR qPCR Master Mix (Vazyme, China) on the QuantStudio 6 Flex (Applied Biosystems, USA). The qPCR data were generated with QuantStudio Real-Time PCR system software. The sequences of the primers used in this study are listed in Supplementary information, *Table S4*.

**Adeno-associated virus (AAV) generation and AAV virus injection**

For AAV virus, AAV9-hTNNT2-mRNAi (negative control, NC)-EGFP-WPRE-pA, AAV9-hTNNT2-mRNAi (*Chrna4*)-EGFP-WPRE-pA and AAV9-hTNNT2-mRNAi (Chrna7)-EGFP-WPRE-pA were designed and constructed using standard methods by Taitool Biotech (China). The following shRNA sequences were used for gene knockdown: NC-shRNA: 5′-GTCTCCACGCGCAGTACATTT-3′; *Chrna4*-shRNA: 5′-CCGGAGACTTATCGAATCCAT-3′; *Chrna7*-shRNA: 5′-GCAGTGGAACATGTCTGAGTA-3′. The virus was then injected into adult (6~8 weeks old) male mice through the tail vein (2 × 10^11^ viral particles/mouse). Knockdown efficiency was assessed via Western blot.

**Patch clamp**

A whole-cell patch clamp was applied to record the membrane potentials and currents. Two borosilicate glass microelectrodes were used: one for electrical stimulation and recording with 2–5 MΩ resistances filled with pipette solution and the other for pulsatile administration filled with the bath solution or bath solution containing different pharmacological agents. Pressure in the application pipette channels was adjusted using a PL1–100 picoinjector microinjector (Harvard Apparatus, USA). The stimulation pulses were applied via an EPC-10 amplifier (HEKA, Germany) synchronized with the microinjector for the synchronous administration of the drug puff. The general bath solution contained the following ingredients: 135 mM NaCl, 10 mM glucose, 5.4 mM KCl, 1.8 mM CaCl_2_, 0.3 mM Na_2_HPO_4_, 0.3 mM KH_2_PO_4_, and 10 mM HEPES (pH 7.35, adjusted with NaOH). The standard pipette solution contained the following ingredients: 140 mM KCl, 10 mM EGTA, 10 mM HEPES, 5 mM glucose, and 3 mM Na_2_ATP. The pH was adjusted to 7.2 with KOH. The ACh-induced currents were recorded in the presence of 100 nM tetrodotoxin. The APs or currents were recorded in current clamp or voltage-clamp mode, and all the data were collected via standard recording protocols at room temperature. The data were analyzed with Patchmaster 2.42 (HEKA, Germany).

**ECG recording**

Hearts from the mice were surgically excised and perfused on a Langendorff apparatus with oxygenated Tyrode’s solution. For the ECG recording of the perfused hearts, one of the electrodes was attached to the free wall of the outside of the atria, and the other was screwed into the ventricular apex. The cardiac electrical signals were amplified and recorded with a BioAmp and a PowerLab (AD Instruments, USA). All the data were analyzed manually using LabChart8 software (AD Instruments, USA).

**Animal models**

Myocardial infarction (MI) surgery was performed as described previously^4-6^. Briefly, the mice were anesthetized with isoflurane in an airtight chamber, after which their chest hair was shaved. The chest was then disinfected with iodine, and the mice were placed on a heating pad in the supine position. A midline anterior cervical skin incision was made, and the trachea was exposed by sharp dissection. The trachea was intubated with a 22-G angio-catheter, whose position was confirmed by direct visualization. The mice were ventilated and anesthetized through the trachea and were connected to a respiratory machine (UGO Basile, Italy) and an anesthetic gas machine (Harvard Apparatus, USA). Their breathing was maintained at approximately 120-140 breaths/min. A 1.5-cm vertical left parasternal skin incision was made, the chest cavity was entered through the fourth interspace, and the pericardium was vertically opened. The left anterior descending (LAD) coronary artery was then ligated with a suture at its upper 1/3 location. Ventricular blanching indicated successful occlusion of the vessel. Then, the chest and skin were closed and disinfected with iodine again.

Transverse aortic constriction (TAC) surgery was performed on mice under aseptic conditions as previously described with minor modifications^7^. Through a midline partial sternotomy approach, the aortic arch was isolated between the right innominate and the left common carotid arteries and ligated with a 7-0 silk suture against a 27-G needle. The needle was immediately withdrawn after the ligation, the surgical incision was closed with 4-0 silk suture, and the skin was sealed.

**Ventricular tachycardia/ventricular fibrillation (VT/VF) induction**

VT/VF was induced by using programmed electrical stimulation as previously described, with minor modifications^8^. Briefly, the mice were anesthetized with isoflurane via mask ventilation while maintaining a body temperature of 37°C. Under sterile conditions, access to the jugular vein was gained by a lateral cutaneous incision from the midclavicular to the angulus mandibulae. The jugular vein was dissected from the surrounding tissue and distally ligated. A 1.1-French octapolar electrophysiologic catheter was pushed proximally into the ventricles until the ventricular electrograms could be recorded. The VT/VF was induced by programmed electrical stimulation^9,10^. The pacing was generated by an external stimulator (STG-3008-FA, Multi-Channel Systems, Germany). Pacing was performed at 2× pacing threshold.

**Statistics**

Two-tailed Student’s *t* test or a nonparametric test was used for comparisons between two groups. For comparisons of multiple groups, one-way or two-way ANOVA was used. Categorical data were analyzed by the Fisher’s exact test. p < 0.05 indicated statistical significance. The normally distributed data are shown as the mean ± standard error of the mean (SEM). All the statistical analyses were performed with GraphPad Prism 8.

**Reference**

1.	Wen Q, Gandhi K, Capel RA, Hao G, O'Shea C, Neagu G, et al. Transverse cardiac slicing and optical imaging for analysis of transmural gradients in membrane potential and Ca^2+^ transients in murine heart. *J Physiol* 2018;596:3951-3965. doi: <https://doi.org/10.1113/JP276239>

2.	Tsai W-C, Guo S, Olaopa MA, Field LJ, Yang J, Shen C, et al. Complex arrhythmia syndrome in a knock-in mouse model carrier of the N98S mutation. *Circulation* 2020;142:1937-1955. doi: <https://doi.org/10.1161/CIRCULATIONAHA.120.046450>

3.	Wu Y, Zhou L, Liu H, Duan R, Zhou H, Zhang F, et al. LRP6 downregulation promotes cardiomyocyte proliferation and heart regeneration. *Cell Res* 2021;31:450-462. doi: <https://doi.org/10.1038/s41422-020-00411-7>

4.	Wang Y, Dembowsky K, Chevalier E, Stüve P, Korf-Klingebiel M, Lochner M, et al. C-X-C Motif Chemokine Receptor 4 Blockade Promotes Tissue Repair After Myocardial Infarction by Enhancing Regulatory T Cell Mobilization and Immune-Regulatory Function. *Circulation* 2019;139:1798-1812. doi: <https://doi.org/10.1161/CIRCULATIONAHA.118.036053>

5.	Ahmad F, Lal H, Zhou J, Vagnozzi RJ, Yu JE, Shang X, et al. Cardiomyocyte-specific deletion of Gsk3α mitigates post-myocardial infarction remodeling, contractile dysfunction, and heart failure. *J Am Coll Cardiol* 2014;64:696-706. doi: <https://doi.org/10.1016/j.jacc.2014.04.068>

6.	Lal H, Zhou J, Ahmad F, Zaka R, Vagnozzi RJ, Decaul M, et al. Glycogen synthase kinase-3α limits ischemic injury, cardiac rupture, post-myocardial infarction remodeling and death. *Circulation* 2012;125:65-75. doi: <https://doi.org/10.1161/CIRCULATIONAHA.111.050666>

7.	Kumarapeli ARK, Su H, Huang W, Tang M, Zheng H, Horak KM, et al. Alpha B-crystallin suppresses pressure overload cardiac hypertrophy. *Circ Res* 2008;103:1473-1482. doi: <https://doi.org/10.1161/CIRCRESAHA.108.180117>

8.	Mollenhauer M, Friedrichs K, Lange M, Gesenberg J, Remane L, Kerkenpaß C, et al. Myeloperoxidase mediates postischemic arrhythmogenic ventricular remodeling. *Circ Res* 2017;121:56-70. doi: <https://doi.org/10.1161/CIRCRESAHA.117.310870>

9.	Qu J, Volpicelli FM, Garcia LI, Sandeep N, Zhang J, Márquez-Rosado L, et al. Gap junction remodeling and spironolactone-dependent reverse remodeling in the hypertrophied heart. *Circ Res* 2009;104:365-371. doi: <https://doi.org/10.1161/CIRCRESAHA.108.184044>

10.	Yang X, Wang T, Lin X, Yue X, Wang Q, Wang G, et al. Genetic deletion of Rnd3/RhoE results in mouse heart calcium leakage through upregulation of protein kinase A signaling. *Circ Res* 2015;116. doi: <https://doi.org/10.1161/CIRCRESAHA.116.304940>

**Figure S1 Mouse ventricular cardiomyocytes express the core elements of the endogenous cholinergic system.**

Immunofluorescence staining of main elements of the endogenous cholinergic system in mouse ventricular myocardial sections. Cardiomyocytes were labelled with α-Actinin. Scale bar, 50 μm.

**Figure S2 Human ventricular cardiomyocytes express key components of the endogenous cholinergic system.**

Immunofluorescence staining of main elements of the endogenous cholinergic system in human ventricular myocardial sections. Cardiomyocytes were labelled with α-Actinin. Scale bar, 50 μm.

**Figure S3 Electrical pacing enhances the release of ACh from ventricular cardiomyocytes.**

The ELSIA assay illustrated that electrical pacing at 2 Hz significantly increased the ACh levels in the culture medium containing isolated ventricular cardiomyocytes treated with the vehicle (Veh), but not under the treatment of the inhibitor for ACh synthetase, α-NETA or inhibitor for the vesicle transport of ACh, vesamicol hydrochloride (Vesa). * p < 0.05, calculated by two-way ANOVA; n = 5-7 per group.

**Figure S4 Construction and validation of the α4 nAChR and α7 nAChR knockout mice**

**A**, Schematic diagram showing the construction of *Chrna4* (encoding the α4 nAChR) knockout mice. **B**, Representative Western blot bands and quantitative analysis of α4 nAChR in the ventricles from α4 nAChR knockout and wild-type mice are shown (n = 4 for each group; * p < 0.05, calculated by Student’s *t* test). GAPDH served as a loading control. **C**, Representative M-mode echocardiographic images of the left ventricle and quantitative bar graph of ejection faction are shown. n = 4 for each group. **D**, Schematic diagram showing the construction of *Chrna7* (encoding the α7 nAChR) knockout mice. **E**, Representative Western blot bands and quantitative analysis of α7 nAChR in the ventricles from α7 nAChR knockout and wild-type mice are shown (n = 4 for each group; * p < 0.05, calculated by Student’s *t* test). GAPDH served as a loading control. **F**, Representative M-mode echocardiographic images of the left ventricle and quantitative bar graph of ejection faction are shown. n = 4 for each group.

**Figure S5 Validation of the α4 nAChR and α7 nAChR knockdown efficiency in human induced pluripotent stem cell-derived cardiomyocytes**

**A** and **B**, Knockdown efficiency of siRNA targeting *Chrna4* (encoding the α4 nAChR) and *Chrna7* (encoding the α7 nAChR) in human induced pluripotent stem cell-derived cardiomyocytes. * *p* < 0.05, calculated by Student’s *t* test, n = 4 per groups.

**Figure S6 α4 or α7 nAChR knockout disrupts the conduction of electrical excitation in the ventricle.**

**A**, Representative ECG recordings of wild-type mice (WT), α4 nAChR knockout mice (α4 KO) and α7 nAChR knockout mice (α7 KO) to evaluate the probability of responding to the programmed electrical stimulus. The dropped QRS complexes are highlighted by the red arrow. **B**, A statistical summary of the dropped QRS complexes is shown in the bar graph. * p < 0.05 *vs* WT, calculated by Fisher’s exact test; n = 7-9 per group.

**Figure S7 Generation of cardiomyocyte-specific *Chrna4* or *Chrna7* deficiency mice via AAV9 virus**

**A**, Schematic diagram showing the construction of cardiomyocyte-specific AAV virus encoding *Chrna4* (encoding the α4 nAChR) shRNA. **B**, Representative Western blot showing the protein level of α4 nAChR in the ventricular cardiomyocytes from mice injected with control virus and AAV9-hTNNT2-miRNAi (*Chrna4*)-EGFP. β-ACTIN served as a loading control. **C**, A bar graph showing the knockdown efficiency of *Chrna4* in mice injected AAV virus (n = 4 for each group; * p < 0.05, calculated by Student’s *t* test). **D**, Schematic diagram showing the construction of cardiomyocyte-specific AAV virus encoding *Chrna7* (encoding the α7 nAChR) shRNA. **E**, Representative Western blot showing the protein level of α7 nAChR in the ventricular cardiomyocytes from mice injected with control virus and AAV9-hTNNT2-miRNAi (*Chrna7*)-EGFP. β-ACTIN served as a loading control. **F**, A bar graph showing the knockdown efficiency of *Chrna7* in mice injected AAV virus (n = 4 for each group; * p < 0.05, calculated by Student’s *t* test).

**Figure S8 Cardiomyocyte-specific α4 or α7 nAChR deficiency disrupts the conduction of electrical excitation in the ventricle.**

**A**, Representative activation time maps of hearts isolated from wild-type (WT), cardiomyocyte-specific α4 or α7 nAChR deficiency mice under basal conditions (left). Quantification of the ventricular conduction velocity (CV) is shown in the bar graph (right, * p < 0.05 vs WT, calculated by one-way ANOVA; n = 7-9 per group). **B**, Representative *in vitro* ECG images of WT, cardiomyocyte-specific α4 or α7 nAChR deficiency mice (left). A statistical summary of the QRS duration is shown in the bar graph (right, * p < 0.05 vs WT, calculated by one-way ANOVA; n = 8 per group).

**Figure S9 The conduction of electrical impulses between ventricular cardiomyocytes is regulated by both gap junctions and the endogenous cholinergic system.**

**A**, Representative activation time maps of ventricles perfused with vehicle (Veh), 2 mM 1-heptanol (Hepa) or 10 μM benzethonium chloride (Ben) plus 2 mM Hepa. **B**, Representative ECG recordings of isolated hearts perfused with Veh, 2 mM Hepa or 10 μM Ben plus 2 mM Hepa.

**Figure S10 The endogenous cholinergic system primarily affects the electrical excitation conduction in ventricular cardiomyocytes rather than in Purkinje fibers.**

**A**, Representative images of electrical excitation transmission in rabbit Purkinje fiber-myocardium samples captured by high-resolution optical mapping. The bright-field images alongside the optical mapping images visualize the anatomic structure of the Purkinje fiber and the ventricle. **B**, The pooled data of the conduction velocity (CV) of electrical excitation in either the Purkinje fibers or myocardium are presented (* p < 0.05, calculated by Student’s *t* test; n = 6 per group). Ventricular myocardium, VM; Purkinje fiber, PF; vehicle, Veh; benzethonium chloride, Ben.

**Figure S11 Defects in the endogenous cholinergic system are linked to fatal cardiac arrhythmias in animal models.**

**A** and **B**, Top, representative ECG images of mice after myocardial infarction (MI) or transverse aortic constriction (TAC) surgery followed by ventricular tachycardia/ventricular fibrillation induction through programmed electrical stimulation. Middle, representative Western blot images showing differences in the expression levels of key members of the endogenous cholinergic system in the ventricular myocardium from mice that underwent sham, MI or TAC surgery. Bottom, the bar graph summarizes the expression levels of the key members of the endogenous cholinergic system. * p < 0.05, calculated by one-way ANOVA; n = 4 per group.

**Figure S12 Knockout of α4 nAChRs or α7 nAChRs increases susceptibility to ventricular arrhythmias.**

**A,** Representative ECG recordings of wild-type mice and α4 nAChR knockout mice after programmed electrical stimulation *in vivo*. **B** and **C,** Bar graph shows the ventricular tachycardia/ventricular fibrillation (VT/VF) induction rate and VT/VF duration in wild-type mice and α4 nAChR knockout mice (* p < 0.05, calculated by Fisher’s exact test, n = 6-7 per group). **D,** Representative ECG recordings of wild-type mice and α7 nAChR knockout mice after programmed electrical stimulation *in vivo*. **E** and **F,** Bar graph shows the VT/VF induction rate and VT/VF duration in wild-type mice and α7 nAChR knockout mice (* p < 0.05, calculated by Fisher’s exact test, n = 6-7 per group).

**Figure S13 Activation of nAChRs prevents fatal ventricular arrhythmias.**

**A,** Experimental design for analyzing the effects of the desformylflustrabromine (dFBr) in the prevention of ventricular arrhythmias. **B,** Representative ECG recordings of isolated heart of mice show that 20 Hz pacing protocol successfully induced ventricular tachycardia/ventricular fibrillation (VT/VF) in the vehicle control. However, pre-treatment with dFBr, prevented the occurrence of the VT/VF under the same burst pacing stimulation. Bar graph shows VT/VF induction rate in heart with or without the pre-treatment of dFBr (right). * p < 0.05, calculated by Fisher’s exact test, n = 8 per group. **C,** Representative ECG recording of mice shows that 20 Hz pacing protocol successfully induced VT/VF in the vehicle control. However, pre-treatment with dFBr prevented the occurrence of the VT/VF under the same burst pacing stimulation. Bar graph shows VT/VF induction rate in heart with or without the pre-treatment of dFBr (right). * p < 0.05, calculated by Fisher’s exact test, n = 8 per group.
